# The Nexus of HIV, substance abuse, and mental health among adolescents in Zambia (2021–2023)

**DOI:** 10.4102/jphia.v16i1.1229

**Published:** 2025-04-30

**Authors:** Belia Longwe, Lushomo Hachilensa, Chipwailia Chunga, Kutha Banda, Muchindu Ng’andu, Hilda Shakwelele, Trevor Mwamba, Nsanzya Maambo, Japhet Michelo, Prudence Haimbe, Mable Mweemba

**Affiliations:** 1 Department of Programs, Clinton Health Access Initiative, Lusaka, Zambia; 2 Department of Programs, Women in Global Health Zambia, Lusaka, Zambia; 3 Department of Policy and Planning, Ministry of Health, Lusaka, Zambia; 4 Department of Research, National Health Research Authority, Lusaka, Zambia; 5 Department of Research Coordination, Capacity Building and Knowledge Translation, National Health Research Authority, Lusaka, Zambia; 6 Department of Public Health, Ministry of Health, Lusaka, Zambia

**Keywords:** HIV, incidence, mental health, substance abuse, adolescents, trends

## Abstract

**Background:**

Adolescents in Zambia face interrelated health challenges, including human immunodeficiency virus (HIV), substance abuse and mental health disorders (MHDs). These issues have significant public health implications, as substance abuse and MHDs are known to increase the risk of HIV incidence.

**Aim:**

This study aimed to analyse trends in HIV incidence, substance abuse and MHDs among Zambian adolescents aged 10–19 years from 2021 to 2023.

**Setting:**

Data were retrieved from the Health Management Information System under Zambia’s Ministry of Health, covering all 10 provinces.

**Methods:**

A retrospective analysis of secondary data was conducted using District Health Information Software 2 (DHIS2), the Ministry of Health’s primary data system. Microsoft Excel and Stata were used for descriptive statistics and regression analysis to examine potential associations.

**Results:**

Findings indicate an increase in national HIV incidence rates from 1.89 in 2021 to 1.99 in 2022, before a decrease to 1.73 in 2023. Substance abuse also showed an upward trend, rising from 0.35 to 0.68 per 1000 adolescents. Incidence of MHDs more than doubled from 0.7 in 2021 to 1.54 in 2023, with Lusaka and Northwestern provinces showing the most significant increases. Our linear correlational analysis showed a positive relationship among the key variables.

**Conclusion:**

The results underscore the interconnectedness of HIV, substance abuse and mental health issues among adolescents in Zambia, emphasising the need for integrated interventions.

**Contribution:**

This study contributes valuable insights for policy and programme development, highlighting the need for targeted, holistic approaches in adolescent health services to address these interconnected issues effectively.

## Background

Adolescents and young people represent a growing share of people living with human immunodeficiency virus (HIV) worldwide. According to United Nations International Children’s Emergency Fund (UNICEF), in 2023, about 1 million (680 000–1.3 million) adolescents between the ages of 15 years and 19 years were living with HIV worldwide. Adolescents account for about 3% of all people living with HIV and about 12% of new adult HIV infections. About 840 000 (550 000–1.1 million] (84%) people living with HIV live in sub-Saharan Africa. In addition, 360 000 young people between the ages of 15 years and 24 years were newly infected with HIV, of whom 140 000 were adolescents between the ages of 15 years and 19 years. To compound this, most recent data indicates that only 29% of adolescent girls and 19% of adolescent boys aged 15–19 years in Eastern and Southern Africa, the region most affected by HIV, have been tested for HIV in the past 12 months. If current trends continue, there will still be an anticipated 183 000 annual new HIV infections among adolescents by 2030.^1^

In Zambia, the prevalence of HIV among adolescents and young people aged between 15 years and 24 years was 3.8% in 2021. Among those aged 15–19 years, the prevalence was 2.6% for girls and 1.2% for boys. This was significant decline in prevalence since 2014 within this age category, from 4.8% and 4.1% among the girls and boys, respectively. In addition, the country records approximately 260 000 new HIV infections among 15–24 year old.^2^

On the other hand, substance abuse among adolescents has increasingly become a matter of public health concern requiring urgent intervention. Substance abuse refers to the use of selected substances, including alcohol, tobacco products, drugs, inhalants and other substances that can be consumed, inhaled, injected or otherwise absorbed into the body with possible dependence and other detrimental effects.^3^ Globally, the World Health Organization (WHO) records that drug use disorders were among the top five causes of adolescent morbidity and mortality in 2019. Alcohol and drug use also contribute about 3.5 million deaths each year and, in most cases, lead to disabilities and poor health.^4^ A comprehensive review conducted in sub-Saharan Africa found that the lifetime prevalence of any substance use among young people is approximately 21.0%, with current use estimated at 15.0%. Alcohol was identified as the most commonly used substance, with a lifetime prevalence of 36.2%, followed by cannabis at 11.0%. Regional variations were observed, with Southern Africa reporting the highest lifetime prevalence at 25.0%, followed by East Africa at 22.0% and West Africa at 17.0%.^5^

Further research also showed that more than one quarter of all people aged 15–19 years were estimated to be current drinkers in 2016, amounting to 155 million adolescents, which represented 45.7% of heavy episodic drinkers among those adolescents.^5^ Data according to WHO supported this assertion and estimated that the percentage of current drinkers below the age of 25 years in Zambia increased from 44% in 2014 to 53% in 2018 among adolescents aged 15–19 years.^6^ Also, alcohol and substance abuse has become a concern among adolescents as the vice increases. A Global School-based Student Health Survey (GSHS) conducted in 73 low- and middle-income countries (LMICs) to ascertain prevalence of addictive behaviours among adolescents aged 13–18 years revealed that in relation to regular alcohol use, Zambia had a prevalence rate of 0.09, which was the highest in the region.^7^

In the Zambian context, urban adolescents and young people were more likely to report engaging in sexual intercourse while intoxicated or with an intoxicated partner, with a prevalence of 7%, compared to their rural counterparts, where the rates were 5% among females and 4% among males.^8^ A study by Mungandi et al. in Zambia found that despite intensified alcohol prevention efforts in schools and communities aimed at increasing knowledge, promoting anti-drug attitudes, and encouraging behaviour change, alcohol use among young people aged 15–24 years continued to rise.^9^ This trend persisted despite the existence of the 2018 National Alcohol Policy.^10^ A study by Vinikoor et al.^11^ also highlighted the burden of alcohol use in Zambia and postulates that the vice has negative impact on the control of the HIV epidemic.

Furthermore, a study conducted in Zambia by Nkwemu^12^ highlighted that cultural and social norms hindering open discussion of sexuality issues perpetuates the sexual risky behaviours among adolescents, usually arising from cultural practices leading to stigma and discrimination, pushing adolescents to alcohol and substance abuse as coping mechanisms to stresses of life, among those deemed to have fallen short of society norms.

Another critical and neglected public health issue that is linked to HIV acquisition among adolescents is mental health disorders (MHDs). Mental health disorders account for 16% of the global burden of disease and injury in adolescents,^13^ with half of all MHDs starting by 14 years of age and most being undetected and untreated.^14^ A systematic and meta-analysis review conducted in Africa found the overall prevalence of mental health distress among adolescents to be 27.34%.^15^ In Zambia, the prevalence of mental disorders is approximately 20.0%; however, mental health continues to be an inadequately researched, neglected and underfunded component of primary healthcare. According to Zambia’s 2020 Mental Health Atlas, there was no specific mental health policy targeted at adolescents despite them being the most affected by the vice and only 0.1% of government expenditure is directed towards addressing mental health issues.^16^

Research suggests that there exists a strong relationship between excessive alcohol consumption and elevated levels of HIV prevalence throughout sub-Saharan Africa and that alcohol and drug use are one of the main avenues placing youths at higher risk of HIV infection.^17^ In addition, psychiatric morbidity is commonly co-occurring with substance abuse and HIV and/or acquired immunodeficiency syndrome (AIDS). The relationship is bi-directional, meaning individuals suffering from mental distress may drink or abuse substances more and vice versa.^18^ Another study supports this assertion and suggests that initiation of alcohol usage has been correlated with mental disorders and results of the study revealed that symptoms of depression were more prevalent in adolescents who had tried alcohol compared with adolescents who had never tried alcohol.^19^

Healthcare services for HIV, substance abuse and mental health in Zambia are structured across different levels to enhance accessibility. Primary healthcare facilities focus on prevention, early detection and basic interventions, while community-level services provide rehabilitation for reintegration and recovery. First-level to tertiary facilities offer specialised curative and rehabilitation services for complex cases. HIV services are available in all health facilities, guided by national protocols to ensure standardised care. However, challenges such as stigma, resource limitations and geographical disparities affect accessibility, particularly in rural areas. Strengthening the integration of these services remains essential for improving health outcomes.^20^

The link between the HIV risk sexual behaviours and MHDs in adolescent population remains largely unexplored. HIV infection among adolescents with MHDs remains an important public health problem, but existing research is very scanty. Thus, this study aimed to determine the trends and nexus in incidence of HIV, substance abuse and MHDs among adolescents in Zambia from 2021 to 2023. Findings of the study will also help to ascertain the extent to which adolescents are indulging in alcohol abuse and how this interplays with HIV and MHDs. These findings will also help to inform policy direction and programme planning around adolescent healthcare by ensuring that interventions are put in place to reduce the levels of alcohol consumption, which will consequently help to reduce their risk of co-occurring morbidity of HIV and MHDs to ensure that they have better health outcomes as they are a population of interest in the healthcare provision.

## Research methods and design

### Study design

This was a secondary data analysis using retrospective programme data on HIV incidence, substance abuse and MHDs from 2021 to 2023 from the Health’s Management Information System (HMIS).

### Setting

This analysis utilised secondary data from the HMIS for the period 2021–2023 across all the 10 provinces of Zambia in facilities that provide HIV, substance abuse and mental healthcare services. The 10 provinces are a mix of rural and urban facilities from primary healthcare to tertiary level care. The different levels of health facilities are surrounded on the one hand by cosmopolitan community catchments, densely populated informal settlements prominent for unregulated trading of alcohol while on the other hand we find sparsely populated residential areas for middle- and high-class populations.

### Study population

The analysis *focussed*> on adolescents aged 10–19 years across the 10 provinces of Zambia.

### Data collection

The analysis used secondary data from District Health Information Software 2 (DHIS2), which is the Ministry of Health’s primary data source. Data were mined by officials from the Monitoring and Evaluation (M&E) Unit at the Ministry. Data were extracted for the period from 2021 to 2023, covering health facilities from the primary health facilities to tertiary level. Specifically, data on HIV incidence, substance abuse and mental health disorders among adolescents aged 10–19 years were extracted. Data elements related to HIV, substance abuse and mental health disorders categorised by age group were extracted from DHIS2 using an analytical tool called Data Visualizer. The data were filtered by age group and period of focus. This was followed by exporting the data to Microsoft Excel for further analysis. In addition, we further collected population data from the Zambia Statistics Agency which were used as populations at risk for the incidence calculations.

### Data validity

To ensure data validity in DHIS2, several strategies were used such as checking for completeness and accuracy of data in the system. At the point of extracting the data, report completeness was at 99.9%. Automated integrity checks and cross-field validations were also employed to check for inconsistencies.

### Data analysis

The extracted data were analysed using both Microsoft Excel and Stata. Descriptive analysis of key variables, including HIV, substance abuse and mental health disorder, was performed in Microsoft Excel to summarise trends and distributions. Incidence rates were calculated using the standard formula adopted by the Ministry of Health, dividing the number of new cases by the population at risk within each year of our focus (2021–2023) and multiplying by a standard factor of 1000 for comparability.

Stata was utilised to conduct a Pearson correlation analysis among the three key variables, identifying potential associations among these variables and exploring their interrelationships.

### Ethical considerations

Approval for a waiver was obtained from the National Health Research Authority because the analysis did not utilise primary data. Permission was also sought from the Ministry of Health to access the data from the DHIS2. Confidentiality and anonymity were also maintained because the mined data do not have client identifiers and were presented in aggregate form.

## Results

**FIGURE 1:**
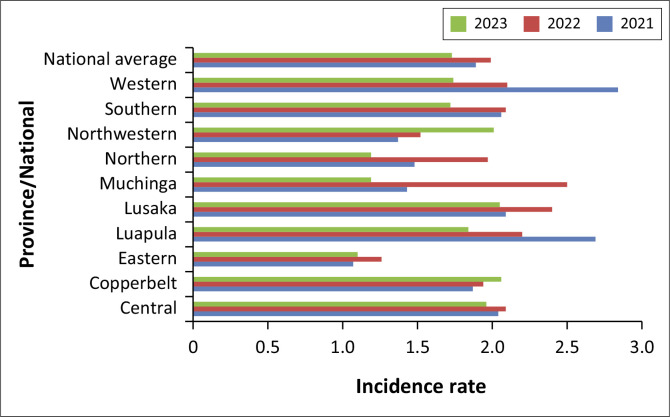
Human immunodeficiency viruses incidence among adolescents (10–19 years) by year (2021–2023).

Our analysis found that the national average of new HIV cases per 1000 adolescents was fluctuating with an increase from 1.89 in 2021 to 1.99 in 2022 and then a slight decrease to 1.73 in 2023 as shown in Figure 1. At provincial level, Lusaka consistently had the highest rates, peaking at 2.40 in 2022 and slightly declining to 2.05 in 2023. Northwestern province also saw an increase in incidence, from 1.37 in 2021 to 2.01 in 2023. Copperbelt showed a steady increase from 1.87 in 2021 to 2.06 in 2023, while new cases in Luapula decreased significantly from 2.69 in 2021 to 1.84 in 2023. Muchinga experienced a notable increase in 2022 with a rate of 2.50 before falling to 1.19 in 2023. Eastern province had relatively stable lower rates, with a minor rise in 2022 and a slight drop in 2023. Western province, despite a high rate of 2.84 in 2021, saw a decrease of 1.74 by 2023. Northwestern province also witnessed an increase in incidence, from 1.37 in 2021 to 2.01 in 2023.

The period 2021 to 2023 showed a notable increase in substance incidence across the provinces, with an overall rise from 0.35 in 2021 to 0.54 in 2022 and reaching 0.68 in 2023 per 1000 adolescents. Significant increases occurred in Lusaka, which went from 0.41 in 2021 to 1.25 in 2023, and Northwestern, which surged from 0.58 in 2021 to 1.65 in 2023. Copperbelt also saw a significant increase from 0.55 in 2021 to 0.86 in 2022 before slightly decreasing to 0.76 in 2023.

**FIGURE 2:**
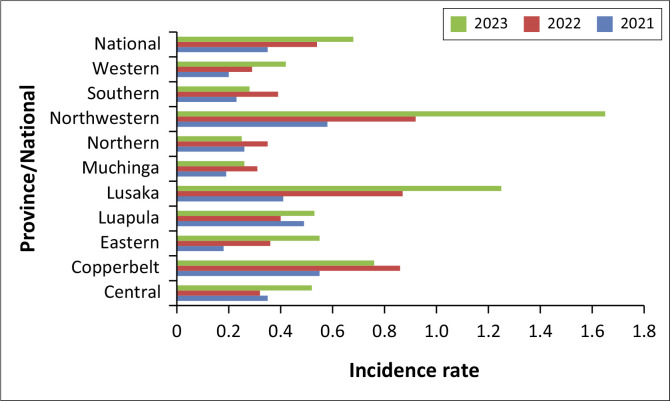
Substance abuse incidence among adolescents (10–19 years) by year.

Other provinces also showed varying patterns. Central province showed an increase in substance incidence from 0.35 in 2021 to 0.52 in 2023, while in Eastern province it rose from 0.18 in 2021 to 0.55 in 2023. Luapula’s rate fluctuated but ended higher at 0.53 in 2023, and Muchinga experienced a decrease from 0.31 in 2022 to 0.26 in 2023. Substance incidence in Northern province decreased to 0.25 in 2023 after an initial rise, and Southern province saw a reduction to 0.28 in 2023. In Western province substance incidence consistently increased each year, reaching 0.42 in 2023 as demonstrated in Figure 2.

**FIGURE 3:**
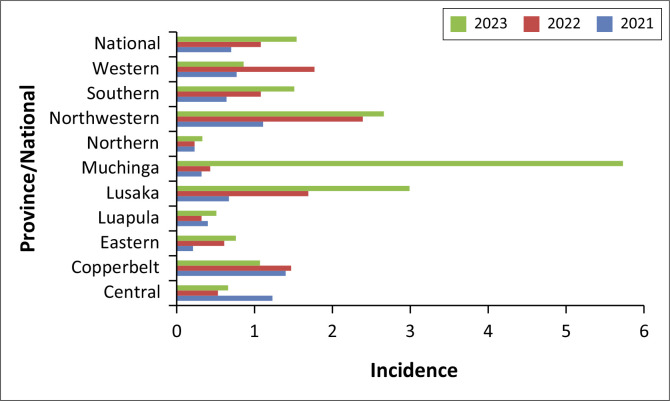
Mental health disorder incidence among adolescents (10–19 years) by year.

We found variations in mental health incidences among adolescents across different provinces from 2021 to 2023 as shown in Figure 3. Nationally, the incidence rate of mental health issues increased from 0.7 in 2021 to 1.54 in 2023, with a corresponding increase in the total number of reported cases from 2914 to 6825.

Notable trends at provincial level included an increase in the proportion of adolescents with a MHD in Lusaka, which rose from 0.67 in 2021 to 2.99 in 2023. Muchinga province also had a significant incidence increase from 0.32 to 5.73 over the same period. On the contrary, the Copperbelt and Luapula regions demonstrated more stable to moderate increases, with Copperbelt experiencing a slight decline. Eastern and Southern regions exhibited steady increase, while Western region showed some fluctuations. Northern province remained relatively stable with minimal changes over the observed period.

**FIGURE 4:**
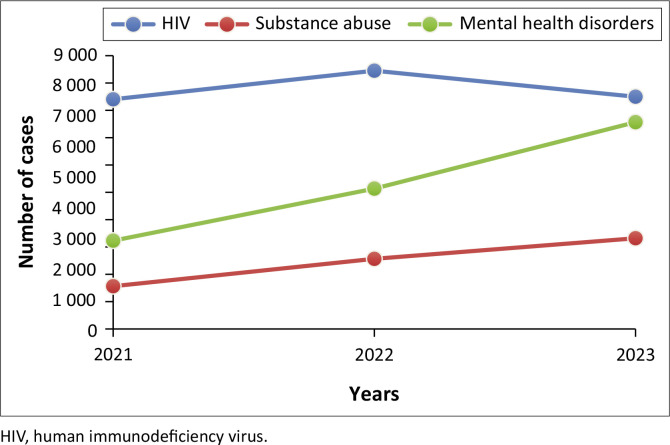
Trends in substance abuse, mental health disorders and human immunodeficiency virus from 2021 to 2023.

The trend analysis in Figure 4 shows that HIV incidences exhibit a notable general increase, marked by a significant peak in 2022 followed by a slight decline in 2023. Both mental health disorders and substance abuse show an upward trend between 2021 and 2023.

**FIGURE 5:**
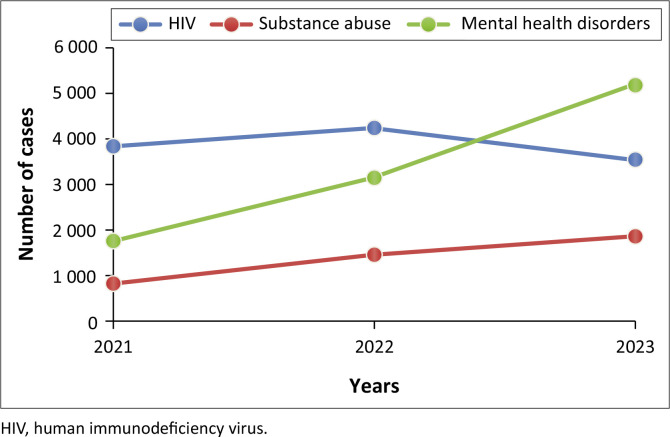
Urban facility trend analysis in substance abuse, mental health disorders and human immunodeficiency virus from 2021 to 2023.

Figure 5 shows substance abuse, mental health disorder and HIV trends in urban facilities within a 3-year period. The trends show that HIV cases increased significantly from 2021 to 2022, peaking at above 4000 cases, and then slightly decreasing to 3500 in 2023. Substance abuse cases steadily increased each year, from approximately 1000 in 2021 to 2000 in 2023. Mental health issues rose from 2000 in 2021 to 3000 in 2022 but further saw a significant increase to over 5000 cases in 2023.

**FIGURE 6:**
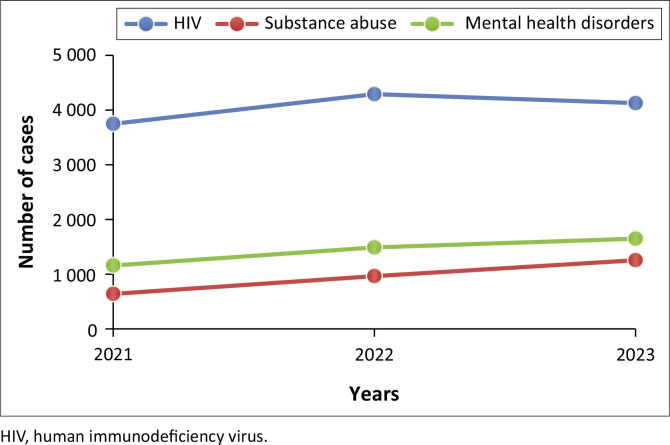
Rural facility trend analysis in substance abuse, mental health disorders and human immunodeficiency virus from 2021 to 2023.

Figure 6 depicting the trends for HIV cases in rural areas shows that the HIV cases increased significantly from 3700 in 2021 to 4200 in 2022, then decreased to 4100 in 2023. The figure further shows that substance abuse cases have steadily increased each year from 600 in 2021 to over 1600 in 2023 and mental health disorders have also increased from 1000 in 2021 to 1600 in 2023.

In our analysis of aggregated data over a 3-year period, we found that all three variables – HIV cases, substance abuse and mental health cases were strongly interrelated. Notably, the association between substance abuse and mental health cases was the most pronounced, with a Pearson correlation of *r* = 0.91, indicating a positive linear relationship. This suggests that individuals with substance abuse problems are highly likely to experience mental health issues. In addition, HIV cases were positively correlated with both substance abuse (*r* = 0.69) and mental health cases (*r* = 0.68). Meaning that as substance abuse increases, the number of HIV cases also tends to increase, this relationship is similar between mental health and HIV incidence also. These results highlight the significant interplay among HIV, substance abuse and mental health issues, pointing towards the potential benefits of integrated intervention strategies (see Table 1).

**TABLE 1:** Pearson correlation among the key variables (linear relationship).

Variables	Variables	Correlation coefficient
HIV cases	Substance abuse	0.69
HIV cases	Mental health	0.68
Substance abuse	Mental health	0.91

HIV, human immunodeficiency virus.

## Discussion

The findings from our analysis reveal a multifaceted landscape of HIV, mental health disorders and substance abuse in Zambia from 2021 to 2023. This section will delve into the trends observed, the interrelationships among the variables, the implications for public health strategies and recommendations for future research.

### Trends in HIV incidence

Several studies have shown fluctuating trends in HIV incidence among adolescents. While some regions have shown steady reductions in new infections, other regions continue to face multiple challenges. Factors such as changes in sexual behaviour, access to antiretroviral therapy (ART) and various public health interventions have all contributed to the trends and fluctuations in HIV incidence among adolescents. Globally, it has been reported that the number of new infections dropped by approximately 30% from 2010 to 2022.^21^ Similarly, results of this analysis also showed a reduction in overall HIV incidence with a few isolated cases of minimal increases in certain regions. Overall, Zambia recorded a reduction in HIV incidence from 1.89 in 2021 to 1.73 in 2023 representing an 8.4% reduction rate.

In addition, rural urban disparities in HIV incidence were a major finding of this analysis. The results showed higher incidence in urban provinces such as Lusaka and Copperbelt provinces compared to rural provinces. Particularly, Lusaka consistently had the highest rates, peaking at 2.40 in 2022 before slightly declining to 2.05 in 2023. This trend underscores the need for targeted interventions in urban areas that have high HIV incidence. Another noteworthy finding was the drastic increase in Northwestern province from 1.37 to 2.01 across the reference period. While the sudden rise in HIV infections can potentially be attributed to the urbanisation that has resulted from the increased mining activities in the province,^22^ the occurrence still warrants further investigation for effective mitigation measures to be put in place.

Conversely, rural provinces such as Luapula, Muchinga and Western province showed significant and consistent decreases in HIV incidence. According to the Global AIDS, the incidence of HIV in urban areas can be attributed to a number of factors such as concentrated populations, higher mobility and the presence of social networks that may facilitate the indulgence into risky sexual behaviour such as unprotected sex and drug use.^23^

### Substance abuse patterns

Substance abuse among adolescents also saw an increasing trend from 0.35 in 2021 to 0.68 in 2023 in Zambia. Most studies suggest that early (12–14 years old) to late (15–17 years old) adolescence is a critical risk period for the initiation of substance use.^24^ Some studies have however demonstrated that many young people use drugs to cope with the social and psychological challenges that they may experience during different phases of their development from adolescence to young adulthood.^25^

Lusaka and Northwestern provinces showed significant increases, indicating hotspots for substance abuse that require focussed interventions. These findings are in line with studies that have highlighted urban facilities as hotspots for substance abuse because of higher availability of substances and peer influence.^2^ Urbanisation has been shown to increase substance abuse in adolescents.^24^ Social media accessibility has also been shown to increase substance abuse among adolescents. The media is a powerful influence on social norms and other messages that are favourable to substance use by adolescents because they spend a great deal of time using the Internet, messaging services and social media, on smartphones, as well as being entertained by television, movies and other media. Media portrayals of substance use as glamorous, fun and relaxing all contribute to the initiation and continued use of psychoactive substances among young people. In essence, certain media messages can make substance use appear to be normative and can alter attitudes about the safety of substance use. Social media has been repeatedly linked to the initiation of substance use.^26^

This validates what has been observed in this study with urban facilities showing higher prevalence of substance abuse. Other provinces displayed varying patterns, with some such as Central and Eastern showing consistent increases, while others such as Muchinga and Northern showed minimal changes over the observed period. These variations point to the complexity of substance abuse dynamics and the necessity for tailored approaches in different regions. The consistent rise in substance abuse across both urban and rural areas suggests widespread ongoing initiation of substance abuse amid challenges in addressing this issue. Adolescents in urban areas as per the findings from urban facilities with higher population densities and greater availability of substances, may be particularly more vulnerable.

### Mental health trends

Mental health issues among adolescents have increasingly become a matter of public health concern. Results of this analysis revealed that at national level, mental health incidence exhibited a stark increase from 0.7 in 2021 to 1.71 in 2023. The corresponding absolute numbers of reported cases rose from 2914 to 6825 within the said period. Particularly, Lusaka’s drastic rise from 0.67 to 2.99 and Muchinga’s surge from 0.32 to 5.73 are of major concern. These results are consistent with global trends indicating rising mental health issues among adolescents, which are believed to be exacerbated by factors such as social media use, academic pressure and coronavirus disease 2019 (COVID-19) pandemic.^27^ Another study that focussed on the impact of *HIV and AIDS* on the mental health of adolescents in sub-Saharan Africa revealed that adolescents living with HIV are more susceptible to MHDs such as depression, anxiety and social stigma.^14^

However, our analysis also determined that the link between MHDs and trends in HIV is bi-directional. A prospective cohort of young people in Eastern Cape, South Africa, were investigated to discern whether depressive symptomatology was associated with risky sexual behaviour. Results showed that individuals with depressive symptoms were more likely to report lifetime intimate partner violence. In females, depressive symptomatology was associated with transactional sex and having dated an older partner. However, males with depressive symptoms were more likely to report ever having had transactional sex and perpetration of rape. It further revealed that males were also less likely to report correct condom use at last sex^28^ thus increasing the risk of HIV infection. The observed trends and study findings therefore imply an urgent need for enhanced interventions in the provision of mental health services at various levels of society.

### Interrelationship between variables

Our analysis of aggregated data over a 3-year period revealed a strong interrelationship among HIV cases, substance abuse and MHDs among adolescents. The association between substance abuse and mental health cases was the most pronounced, with a Pearson correlation of 0.91, indicating a strong positive linear relationship. This suggests that adolescents struggling with substance abuse are highly likely to experience mental health issues. In addition, HIV cases were positively correlated with both substance abuse (*r* = 0.69) and MHDs (*r* = 0.68), highlighting a significant interplay between these factors. These findings suggest that as substance abuse increases, the number of HIV cases also tends to rise, a trend similarly observed between MHDs and HIV incidence.

Previous research has shown that substance abuse can lead to risky sexual behaviour, increasing the risk of HIV transmission as demonstrated by results from a study performed by Musuka et al., which suggested a strong association between alcohol consumption and HIV positivity (*p* < 0.001).^29^ This attests to other observations that alcohol consumption may increase risk-taking behaviours among individuals who may not have taken the same risk when they are sober, which potentially exposes them to HIV infection. Sex under the influence of alcohol was associated with a greater likelihood of paying for sex, use of physical force to have sex and unprotected sex. Alcohol consumption has also been suggested to increase the risk of becoming infected with HIV through its suppressive effects on the immune system.^30^

In addition, MHDs can impair judgement and lead to behaviours that increase HIV risk. The strong comorbidity between HIV and MHDs is well-documented with depression symptoms estimated to affect between 12% and 60% of people living with HIV,^31^ although most studies focus on adult populations. Findings from this analysis pertaining to an increase in mental health cases being associated with a rise in HIV are similar to what other studies have also found. A study carried out in Zambia and Zimbabwe found that adolescents with high depressive symptomatology were more likely to report behaviours that placed them at risk of HIV infection compared to those who reported no symptoms.^32^

Mental health disorders can hinder the motivation needed to engage in effective HIV risk-reduction behaviours. The presence of MHDs may potentially contribute to less utilisation of prevention efforts. Overly symptoms of depression and other MHDs should be considered as potential markers of increased HIV risk and this association can be causal.^15^ These findings underscore the interconnectedness of these health issues and the importance of integrated intervention strategies that respond wholistically to adolescents.

### Strengths and limitations

The strength of our analysis is its comprehensive use of national data, drawing on data from all 10 provinces of Zambia to provide a nationally representative perspective on HIV, alcohol abuse and MHDs among adolescents. In addition, the study used secondary data, which minimised potential for biases that arise from primary data collection.

This analysis is not without limitations, including potential underreporting and a lack of reporting by some facilities. In addition, the data concerning our variables of interest lacked uniformity in how the adolescent age bands were disaggregated and hence did not allow for comprehensive comparability. Lastly, the study does not cover other explanatory factors that could explain the observed trends that otherwise could be collected using primary data methods; it is possible that the trends and associated relationships is as a result of other cofounding factors.

### Recommendations

Based on the findings, the following policy recommendations are proposed:

- Integrated Health Services: Developing integrated health services that address HIV, mental health issues and substance abuse concurrently can improve outcomes and reduce the burden on adolescents.
- School-Based Health Programmes: Integrating mental health education, substance abuse prevention and HIV education into school curricula can promote early awareness and equip adolescents with coping skills.
- Task-Shifting and Capacity Building: Training community health workers and primary healthcare providers to deliver basic mental health and substance abuse services can help bridge gaps in specialised care, especially in rural areas.
- Community Support Networks: Strengthening peer-support groups, faith-based organisations and family engagement programmes can provide a supportive environment for adolescents dealing with mental health and substance use issues.
- Leveraging Digital Health Solutions: Using mobile health (mHealth) platforms, telemedicine, and SMS-based counselling services can improve access to mental health and HIV-related support, particularly for adolescents in remote areas.
- Targeted Interventions: Focussed interventions in high-incidence areas, particularly urban centres such as Lusaka, can help address the specific needs of these populations.
- Public Awareness Campaigns: Increasing public awareness about the interconnectedness of HIV, mental health issues and substance abuse can help in reducing stigma and promoting health-seeking behaviours.
- Increased awareness to reduce stigma surrounding mental health illnesses as well as expanded investment in accessible and affordable healthcare services that solely focus on the diagnosis and treatment of MHDs especially in LMICs.

## Conclusion

This analysis provides critical insights into the trends and interrelationships among HIV, mental health issues and substance abuse among adolescents in Zambia, revealing that rising rates in substance abuse and MHDs correlate significantly with increased HIV incidence. Urban areas, particularly Lusaka, show higher prevalence rates, likely because of unique socioeconomic pressures and influences.

The findings emphasise the need for integrated health interventions that address HIV, substance abuse and mental health issues within primary healthcare settings. Strengthening surveillance systems to monitor trends, expanding youth-friendly and community-based services and training healthcare providers in integrated care can improve early intervention and treatment. In addition, school-based health programmes, public awareness campaigns to reduce stigma and increased investment in accessible mental health services – particularly in rural and underserved areas – are critical for improving adolescent health outcomes. Engaging educators, community leaders and peer support networks will further enhance service delivery and encourage health-seeking behaviours.
